# Medicarpin Increases Antioxidant Genes by Inducing NRF2 Transcriptional Level in HeLa Cells

**DOI:** 10.3390/antiox11020421

**Published:** 2022-02-18

**Authors:** Jung-Hwan Kim, Dong-Min Kang, Young-Jin Cho, Jin-Won Hyun, Mi-Jeong Ahn

**Affiliations:** 1 Department of Pharmacology, School of Medicine, Institute of Health Sciences, Gyeongsang National University, Jinju 52727, Korea; 2 College of Pharmacy and Research Institute of Pharmaceutical Sciences, Gyeongsang National University, Jinju 52828, Korea; kdm7105@gnu.ac.kr (D.-M.K.); amj5812@gnu.ac.kr (M.-J.A.); 3 Department of Premedicine, College of Medicine, Gyeongsang National University, Jinju 52828, Korea; yjkai21@gnu.ac.kr; 4 Department of Biochemistry, School of Medicine, Jeju National University, Jeju 63243, Korea; jinwonh@jejunu.ac.kr

**Keywords:** HO-1, *Robinia pseudoacacia* L., antioxidant, ubiquitination, chemoprevention

## Abstract

The nuclear factor erythroid-derived 2-related factor 2 (NRF2) plays a pivotal role in the regulation of genes involved in oxidative stress and drug detoxification. Therefore, it is important to find NRF2 inducers to protect cells from excessive oxidative damage. Here, we investigated the effect of medicarpin isolated from the root of *Robinia pseudoacacia* L. on the activity of NRF2 in HeLa cells. Medicarpin significantly induced the antioxidant response elements (ARE)-luciferase activity in a concentration-dependent manner. Furthermore, medicarpin not only induced *HO-1*, *GCLC*, and *NQO1* mRNA by translocating NRF2 to the nucleus but also induced the mRNA level of *NRF2*. To verify the NRF2 induction mechanism by medicarpin, ~2 kb of NRF2 promoter-luciferase assay was executed. As a result, medicarpin significantly induced NRF2-luciferase activity. Moreover, medicarpin strongly inhibited the ubiquitin-dependent proteasomal degradation of NRF2. Thus, medicarpin might protect cells by promoting the *NRF2* transcriptional activity.

## 1. Introduction

Nuclear factor erythroid 2-related factor 2 (NRF2) is a key transcription factor for the expression of genes responsible for anti-oxidative stress and drug detoxification. The role of NRF2 has been implicated in many stress-induced pathophysiological conditions, such as age-related diseases, inflammation, neurodegenerative, metabolic disorders, and various cancers [1,2,3,4].

Under normal conditions, NRF2 undergoes proteasomal degradation by a ubiquitinoylation process in the presence of kelch-like ECH associated protein 1 (KEAP1). However, under certain stimulations by oxidative stress, electrophilic stress, or various natural chemicals, NRF2 can be stabilized and translocated into the nucleus and triggers induction of NRF2 target genes [5,6,7,8,9,10,11,12].

The major function of NRF2 is the induction of antioxidant enzymes, such as heme-oxygenase-1 (HO-1), glutamate-cysteine ligase catalytic subunit (GCLC), and NAD(P)H: quinine oxidoreductase-1 (NQO-1). Among these, HO-1 has been used as a hallmark as an NRF2 target gene and a major antioxidant enzyme for protecting cells from oxidative stress and inflammation. The expression of these genes can be controlled by NRF2 through interactions with antioxidant response elements (AREs) located in promoters [13,14]. 

To control the NRF2 activity for the induction of its target genes, many signaling molecules are identified. KEAP1 is a representative negative factor responsible for the ubiquitin-dependent proteasomal degradation of NRF2 [15]. In addition, phosphatidylinositol 3-kinase (PI3K)/Akt, cyclic-AMP-activated protein kinase α (AMPKα) [16,17], and IQGAP1 [6] are known as positive factors for NRF2 activity.

It has been reported that various natural substances can induce the NRF2 activity through different molecular pathways by phosphorylating the serine/threonine residues of mitogen-activated protein kinases (MAPKs), cAMP-activated protein kinase (AMPK), AKT, and protein kinase C (PKC) [18,19,20,21] as well as inhibiting the ubiquitin-dependent proteasomal degradation of NRF2 [22,23].

Studies have been conducted looking for ARE inducers in naturally occurring chemicals or plant extracts to utilize beneficial antioxidants to maintain cellular health. OxiCyan^®^, a phytocomplex of bilberry and spirulina, might be an example of an ARE/NRF2 activator in the HepG2 cells [24]. Additionally, ARE/NRF2 inducers can be applied to animal experiments by designing for a specific pathophysiological condition. In an animal study, rosuvastatin was investigated for the effect on high salt and cholesterol diet (HSCD)-induced cognitive impairment in the rats by showing the role of the ARE/NRF2 pathway [25].

*Robinia pseudoacacia* L. (*Fabaceae*), known as black locust, is one of the most common exotics in Europe, North America, and Asia and is used as a medicinal plant for a laxative, antispasmodic, and diuretic [26]. Medicarpin, a natural pterocarpan, has been reported to have various beneficial biological functions in the inhibition of osteoclastogenesis, stimulation of bone regeneration, induction of apoptosis, and induction of lipolysis in adipocytes [27,28,29,30]. Here, we obtained medicarpin from the root of *R. pseudoacacia* and examined the effect on NRF2 activity.

## 2. Materials and Methods

### 2.1. Chemicals

Medicarpin was isolated from the root of *Robinia pseudoacacia* L. obtained in Korea, and the chemical structure was identified by comparing the EI-MS, ^1^H-, and ^13^C-NMR spectroscopic data with the published ones. Anti-NRF2 (ab137550) and anti-HO-1 (ab68477) antibodies were obtained from Abcam (Cambridge, MA, USA). Antibodies against Lamin A/C, GFP, and GAPDH (sc-25778) were purchased from Santa Cruz Biotechnology (Santa Cruz, CA, USA).

### 2.2. Cell Culture

HeLa cells were obtained from American Type Culture Collection (ATCC, Manassas, VA, USA) and maintained in RPMI 1640 medium containing 10% fetal bovine serum and antibiotic-antimycotic (100 units/mL of penicillin, 100 µg/mL of streptomycin, and 0.25 µg/mL of amphotericin B) in a humidified incubator at 37 °C, 5% CO_2_, and 95% air. Cells were grown at 60–70% confluence for sub-culturing and all experiments.

### 2.3. Cell Toxicity Assay

Cytotoxic effect of medicarpin was performed using MTT assay on HeLa cells, as previously described [31]. Briefly, cells were seeded in 48-well plates and treated with different doses of medicarpin (0–100 μM) for 24 h. Then, 20 μL of MTT stock solution (5 mg/mL) was added to each well and followed an additional 2 h incubation. Crystalized formazan in the cells was dissolved by adding DMSO after removing the medium. The intensity of the purple color of formazan was analyzed by reading the absorbance at 570 nm using a plate reader (Varioskan^TM^ LUX, Thermo Scientific^TM^, Waltham, MA, USA).

### 2.4. Cloning

Human NRF2 promoter-luciferase reporter plasmid was constructed by inserting the amplified ~2 Kb sized NRF2 promoter into the modified pGL4.10-Basic vector (Promega, Madison, WI, USA), which contains AscI and PacI restriction enzyme sites in the multicloning sites. For the PCR amplification, the following primers were used: pr-hNrf2-AscI-F2-5′-AAA GGC GCG CCA GCA ATC TGG AGC AAG GTA TCA CAA TTG AC-3′ and Pr-hNrf2-PacI-R2-5′-AAA TTA ATT AAC CCG CGA GAT AAA GAG TTG TTT GCG-3′.

### 2.5. ARE Luciferase Assay

To see the effect of medicarpin on NRF2 activation or *NRF2* transcriptional induction, the ARE-luciferase and ~2 kb *NRF2* promoter assay were executed in HeLa cells using a Dual-Luciferase Reporter Assay kit (Promega, Madison, WI, USA) according to the manufacturer’s instructions. Briefly, cultured cells in 48-well plates were treated with different concentrations of medicarpin (0–100 μM) for 6 h after co-transfection with pGL4.21 3× ARE plasmid (60 ng/well) [22] or pGL4.10- 2 kb-NRF2 promoter plasmid in the presence of pRL-Renilla luciferase control reporter vector (20 ng/well) overnight and then lysed with 100 μL of 1× passive lysis buffer at room temperature. Then, the lysates (10 μL) were used to measure the ARE luciferase activity. The Renilla luciferase activity was used to normalize the ARE-luciferase enzyme activity.

### 2.6. Western Blot Analysis

HeLa cells were cultured in 6-well plates until they reached 60–70% confluency before the addition of medicarpin or DMSO (0.1%) for 24 h with different concentrations as indicated in the figures. Nuclear and cytosolic proteins were fractionated using M-PER buffer, and whole-cell lysates were isolated using RIPA buffer [32]. Protein concentration was determined by reading absorbance at 570 nm using BCA reagent (Thermo Scientific, Waltham, MA, USA). Total proteins (30 µg) were separated on a gradient SDS-polyacrylamide gel (4–20%) and transferred onto a nitrocellulose membrane using the Trans-Blot Turbo system (Bio-Rad, Hercules, CA, USA). After membrane blocking with 5% non-fat dry milk in PBS buffer containing 0.1% Tween-20 for 1 h, the primary antibodies were incubated at 4 °C overnight, followed by incubation with horseradish peroxide-conjugated secondary antibodies for 1 h. Protein signals were visualized using Bio-Rad ECL substrate solution under the ChemiDoc System (Bio-Rad, Hercules, CA, USA).

### 2.7. Real-Time PCR Analysis 

Total RNA was isolated from the cells using TRIzol reagent (Invitrogen, Carlsbad, CA, USA) according to the manufacturer’s instructions. Next, cDNA was synthesized with 1 µg of total RNA using the qScript cDNA Synthesis kit (QuantaBio, Beverly, MA, USA). The PCR reaction was performed using PerfeCTa SYBR Green FastMix (QuantaBio, Beverly, MA, USA). Thermocycler conditions were set as follows: initial denaturation at 95 °C for 30 s; amplification for 45 cycles, including denaturation at 95 °C for 5 s and annealing/extension at 60 °C for 10 sec; and cooling at 4 °C for 10 sec using QuantStudio^TM^ 5 (Applied Biosystem^TM^, Waltham, MA, USA). Primer sets are listed in Table 1. 

### 2.8. Protein Stability Assay 

To study the effect of medicarpin on the ubiquitin degradation of NRF2, HeLa cells cultured in 10 cm dishes were transfected with plasmids DNA of pcDNA4-His-Ubi (3.5 μg) and pEGFP-NRF2 (3.5 μg) for 24 h using polyethyleneimine (PEI) reagent and then treated with medicarpin (50 μM) for 6 h. Next, the cell was lysed with RIPA lysis buffer. Whole-cell extract (250 μg) was incubated with 50 μL of the Ni-NTA agarose slurry in 500 μL RIPA buffer for 1 h at 4 °C in a rotary shaker. After washing with RIPA buffer, the beads were resuspended in 2× Laemmli sample buffer. After boiling the samples for 5 min, Western blotting was executed using SDS-PAGE gel. 

### 2.9. Statistical Analysis

Results were presented as the mean ± SD. Statistical analysis was performed using a two-tailed Student’s *t*-test on unpaired data, and *p* < 0.05 was considered statistically significant.

## 3. Results

### 3.1. Medicarpin Increases NRF2 Activity through Are System in HeLa Cells

**Figure 1:**
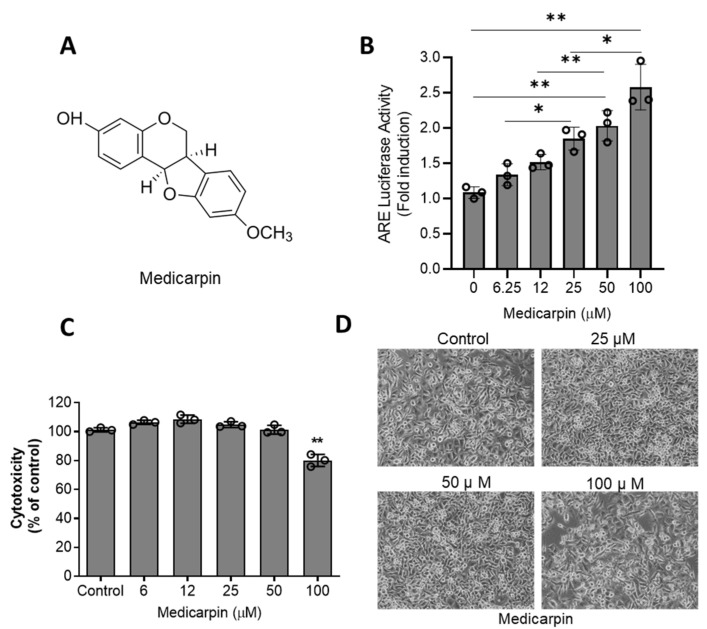
Medicarpin increases the ARE luciferase activity in HeLa cells. (**A**). Chemical structure of medicarpin. (**B**). The ARE-luciferase assay was performed in HeLa cells after treatment with indicated concentrations of medicarpin for 6 h. (**C**). The cytotoxic effect was measured by an MTT assay after treatment with different medicarpin concentrations of for 24 h. (**D**). The representative images of the HeLa cells were pictured after treatment with indicated concentrations of medicarpin for 24 h. The cell images were taken at the same magnification. Experiments were performed in triplicate and repeated three times with similar results. * *p* < 0.05; ** *p* < 0.001.

To examine the effect of medicarpin on NRF2 activity, the ARE luciferase assay was performed in HeLa cells. As a result, medicarpin (Figure 1A) significantly increased the ARE-luciferase activity after a 6 h treatment in a concentration-dependent manner (Figure 1B). Next, to determine the cytotoxicity of MA, an MTT assay was performed after treatment with various concentrations of medicarpin (0–100 μM) for 24 h in HeLa cells. As a result, medicarpin showed a growth inhibitory effect at 100 μM. However, activation of ARE-luciferase at 50 μM was sufficient without a cytotoxic effect (Figure 1B). Cells were imaged after treatment with various concentrations of medicarpin for 24 hours. As shown in Figure 1C, the population of the cells was inhibited only by 100 μM of medicarpin (Figure 1D). 

### 3.2. Medicarpin Increases HO-1 by NRF2 Activation in HeLa Cells

**Figure 2:**
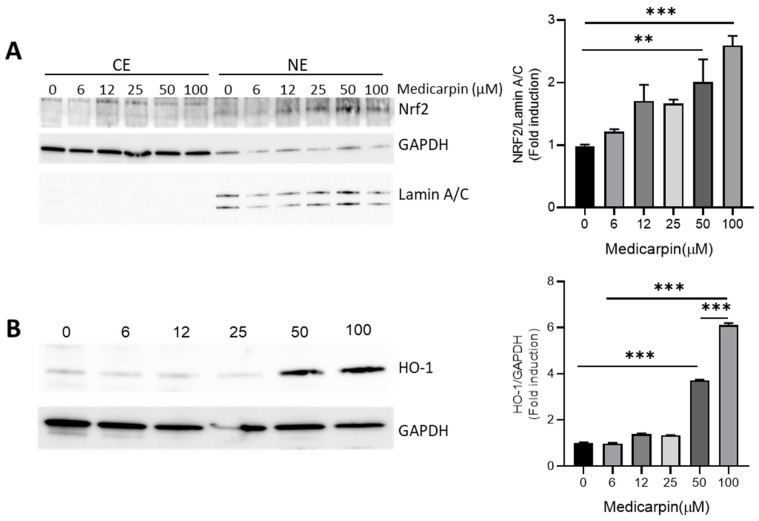
Medicarpin increases nuclear NRF2 accumulation, which results in HO-1 induction in HeLa cells. (**A**). Western blotting data shows nuclear NRF2 level after treatment with different concentrations of medicarpin for 6 h. The densitometrical analysis of nuclear NRF2 is shown in the right panel. (**B**). Western blot analysis using whole cell lysates shows the HO-1 level after treatment with the indicated concentration of medicarpin for 24 h. The densitometrical analysis of HO-1 is shown in the right panel. GAPDH and Lamin A/C were used as cytoplasmic and nuclear markers, respectively. CE, cytoplasmic extract; NE, nuclear extract. The experiments were repeated three times with similar results. ** *p* < 0.001; *** *p* < 0.0001.

To confirm the effect of medicarpin on the NRF2 activity, the accumulation of NRF2 was measured after treatment with different concentrations of medicarpin for 6 h in HeLa cells. The results showed that nuclear accumulation of NRF2 was maximal at 50 μM of medicarpin. Furthermore, HO-1, a representative NRF2 target protein, was strongly increased by the treatment with 50 μM of medicarpin (Figure 2B). Thus, it suggests that medicarpin increases the NRF2 activity, which results in the inductions of NRF2 target genes. 

### 3.3. Medicarpin Increases the Transcriptional Level of NRF2 Target Genes in HeLa Cells

**Figure 3:**
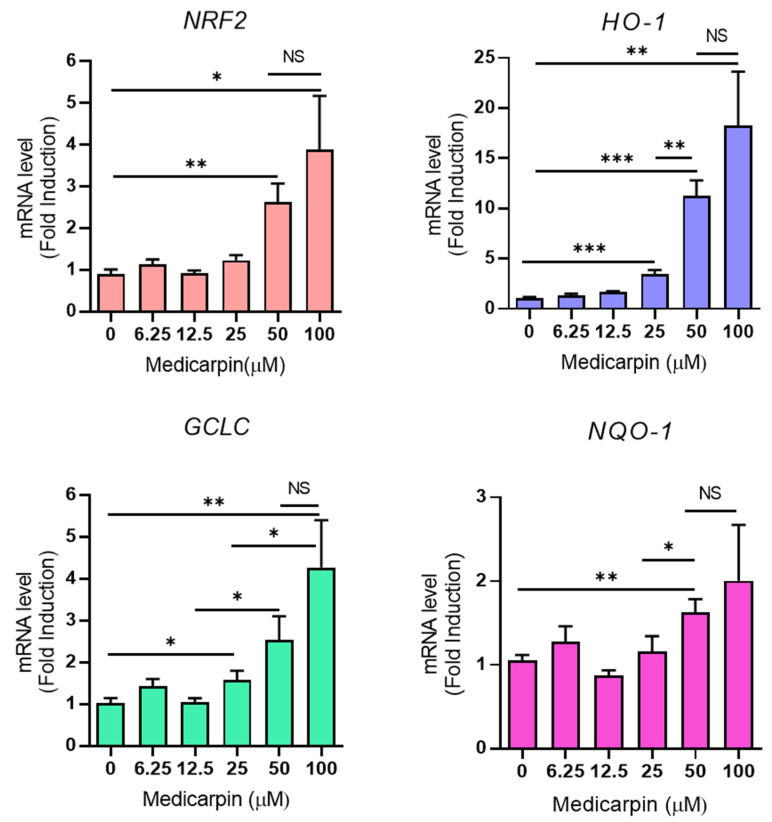
Medicarpin induces the transcriptional level of *NRF2* and NRF2 target genes in HeLa cells. A. Real-time PCR analysis showed the mRNA levels of NRF2, HO-1, GCLC, and NQO-1 after treatment with different concentrations of medicarpin for 24 h in HeLa cells. Experiments were performed in triplicate. * *p* < 0.05; ** *p* < 0.001; *** *p* < 0.0001; NS, not significant.

To explore whether medicarpin could increase the NRF2 target genes, such as *HO-1*, *GCLC*, *NQO*-1 as well as NRF2, the mRNA level of NRF2 target genes was measured using real-time PCR. As result, medicarpin increased the mRNA level of *HO*-1, *GCLC*, and *NQO-1*. Interestingly, medicarpin also increased the mRNA level of *NRF2* (Figure 3). This may suggest that medicarpin can regulate the transcriptional activity of *NRF2*; thus, increased NRF2 can trigger the expressions of NRF2 target genes. 

### 3.4. Medicarpin Increases the Transcriptional Activity NRF2 Gene

**Figure 4:**
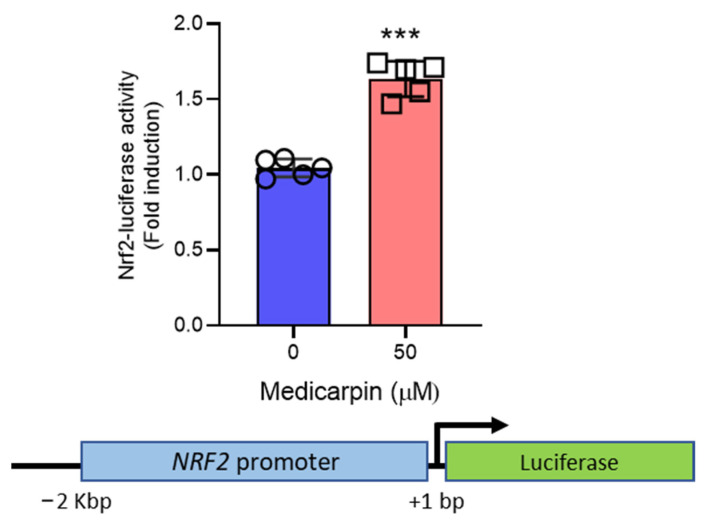
Medicarpin induces the NRF2 transcriptional level in Hela cells. Cells were treated with 50 μM of medicarpin for 6 h in the condition of transfection with an NRF2-promoter luciferase construct. NRF2-luciferase activity was measured using a Dual-Luciferase Reporter Assay kit (Promega) according to the manufacturer’s instructions. Experiments were performed in quintuplicate. *** *p* < 0.0001.

To examine the effect of medicarpin on the transcriptional regulation of *NRF2*, NRF2 promoter (~2 Kbp) was subjected to NRF2-luciferase assay. As result, medicarpin significantly increased the NRF2-luciferase activity at 50 μM (Figure 4). While many existing natural products, such as amentoflavone [33] and juglone [22], contribute to the stabilization of NRF2, medicarpin may function differently by increasing the transcriptional level of *NRF2*, thereby regulating the expression of NRF2 target genes.

### 3.5. Medicarpin Potentiates the NRF2 Stability by Inhibiting Ubiquitin-Mediated Degradation

**Figure 5:**
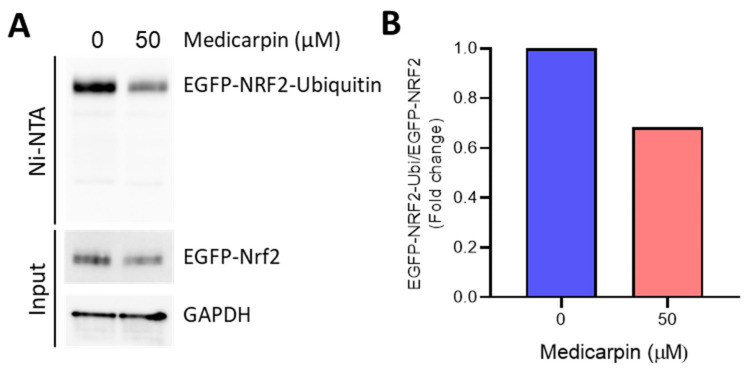
Medicarpin increases the NRF2 stability by inhibiting ubiquitin-mediated proteolysis in HeLa cells. (**A**). Cells were treated with 50 μM of medicarpin for 6 h after co-transfection with pEGFP-NRF2 and pcDNA3.1-His ubiquitin plasmids. Next, cells were lysed with RIPA and His-ubiquitinated proteins were purified using Ni-NTA agarose beads. After washing with RIPA, ubiquitinylated eGFP-NRF2 was visualized by Western blot analysis. (**B**). The relative fold change of ubiquitinylated eGFP-NRF2 was measured using a densitometer from (**A**). Experiments were performed in duplicate.

To explore whether medicarpin could affect the NRF2 stability for further induction of NRF2 target genes since NRF2 protein undergoes the ubiquitin-mediated proteasomal degradation pathway using the KEAP1-Cul3 system [15], we tested the NRF2 stability by using the EGFP-NRF2-Ubiquitin system. The cells were treated with 50 μM of medicarpin for 6 h in the presence of expression of EGFP-NRF2 and His-Ubiquitin. After the Ni-NTA purification step, we observed that the levels of ubiquitinated EGFP-NRF2 were significantly decreased by medicarpin (Figure 5), suggesting that medicarpin also potentiated the NRF2 stability by inhibiting the ubiquitination of EGFP-NRF2. 

## 4. Discussion

In the present study, we provide the effect of medicarpin on NRF2 activation by addressing the ARE luciferase activity, NRF2 nuclear accumulation, and NRF2 transcriptional activity in HeLa cells. Hence, medicarpin fortifies the expression of NRF2 target genes, such as *HO-1*, *NQO-1*, and *GCLC*.

As NRF2 is a major key factor for controlling oxidative stress, many NRF2 signaling pathways have been studied. As an inhibitory pathway, NRF2 signaling can be inhibited by the KEAP1-mediated degradation pathway [15]. However, in the concept of chemoprevention for many pathophysiological symptoms including cancers, activation of NRF2 is recognized as beneficial before cancer modulates the NRF2 activation. Regarding the mechanism of Nrf2 activation, MAPK and PI3K/Akt [16,17], IQGAP1 [6], and RAC3 [32] are involved. 

As medicarpin increased the level of *NRF2* mRNA, targeting the level of NRF2 transcription can be a strategy for the NRF2 activation. To date, the molecular mechanism of NRF2 transcription regulation is not clearly elucidated. Thus, in order to activate NRF2 signaling to control oxidative stress, many possible targeting strategies based on the molecular mechanism-based discovery of novel compounds have been applied. Previously, we reported some naturally occurring compounds corresponding to NRF2 activation. Among the chemicals, amentoflavones [33] and methoxycinnamoyl-α-l-rhamnopyranosyl ester (MCR) [23] were found to be activators of NRF2 by inhibiting ubiquitin-mediated proteasome degradation.

Medicarpin, a naturally occurring phytoestrogen similar to isoflavonoids, is present in a variety of legumes. Although growing evidence shows its various biological effects on bone regeneration, induction of apoptosis, and inhibition of inflammation [27,28,29,30], its molecular mechanism is not clear. However, we speculate that NRF2 induction by medicarpin leads to such biological effects, because inhibition of NRF2 could induce aberrant bone metabolism as well as bone-related inflammation [34,35,36]. Although studies of medicarpin in other diseases, including cancer, are limited, the application of this naturally occurring chemical to other pathophysiological conditions is possible because medicarpin has been shown to be an ARE/NRF2 inducer. Thus, this study suggests that medicarpin can be used for chemoprevention or chemotherapeutic purpose, such as in cancers. 

## 5. Conclusions

In this study, we showed the effect of medicarpin on NRF2 activation by increasing the transcriptional level of NRF2 as well as its stability in HeLa cells. Thus, it is possible that medicarpin could apply to the ARE/NRF2 inducer for chemoprevention.

## Figures and Tables

**Table 1:** Primer sets for real-time PCR.

Gene	Foward	Reverse
***NRF2***	5′-TCT TGC CTC CAA AGT ATG TCA A-3′	5′-ACA CGG TCC ACA GCT CAT C-3′
***HO-1***	5′-GAG TGT AAG GAC CCA TCG GA-3′	5′-GCC AGC AAC AAA GTG CAA G-3′
***NQO-1***	5′-TCC TTT CTT CTT CAA AGC CG-3′	5′-GGA CTG CAC CAG AGC CAT-3′
***GCLC***	5′-CTT TCT CCC CAG ACA GGA CC-3′	5′-CAA GGA CGT TCT CAA GTG GG-3′
***GAPDH***	5′-AAG GTG AAG GTC GGA GTC AA-3′	5′-AAT GAA GGG GTC ATT GAT GG-3′

## Data Availability

Data is contained within the article.
